# Teen Pregnancy Prevention Program Recommendations from Urban and Reservation Northern Plains American Indian Community Members

**DOI:** 10.1080/15546128.2015.1049314

**Published:** 2015-08-28

**Authors:** Tracey R. McMahon, Jessica D. Hanson, Emily R. Griese, DenYelle Baete Kenyon

**Affiliations:** ^a^ Sanford Research, Sioux Falls, SD, USA

**Keywords:** American Indian, teen pregnancy prevention, program recommendations

## Abstract

Despite declines over the past few decades, the United States has one of the highest rates of teen pregnancy compared to other industrialized nations. American Indian youth have experienced higher rates of teen pregnancy compared to the overall population for decades. Although it's known that community and cultural adaptation enhance program effectiveness, few teen pregnancy prevention programs have published on recommendations for adapting these programs to address the specific needs of Northern Plains American Indian youth. We employed a mixed-methods analysis of 24 focus groups and 20 interviews with a combined total of 185 urban and reservation-based American Indian youth and elders, local health care providers, and local school personnel to detail recommendations for the cultural adaptation, content, and implementation of a teen pregnancy prevention program specific to this population. Gender differences and urban /reservation site differences in the types of recommendations offered and the potential reasons for these differences are discussed.

## INTRODUCTION

Although adolescents of all races and ethnicities engage in behaviors such as unprotected sex and substance use that increase their risk for sexually transmitted infections (STIs) and teen pregnancies (Eaton et al., 2012), American Indian/Alaska Native (AI/AN) youth experience disproportionately higher rates of teen pregnancy compared with the overall population (de Ravello, Jones, Tulloch, Taylor, & Doshi, 2014). Furthermore, while the birth rate for U.S. teens aged 15–19 has dropped in 18 of the past 20 years, changes in the annual birth rates for AI/AN teens have been less consistent over the past two decades and have remained notably higher than those of non-Hispanic Whites and Asian/Pacific Islanders (Solomon-Fears, 2013). For example, in 2012, the birth rates for AI/AN teens was 34.9 per 1,000 live births while the national rate reached a historic low of 29.4 and the rate for non-Hispanic White teens was lower yet at 20.5 (Martin, Hamilton, Osterman, Curtin, & Mathew, 2013).

## AMERICAN INDIAN/ALASKA NATIVE SEXUAL RISK BEHAVIORS

Previous findings regarding adolescent sexual behavior also reveal that a greater proportion of urban AI/AN youth engage in sex at younger ages and are less often using any method of contraception compared to the overall population (de Ravello et al., 2014). Specifically, in a study examining the reproductive health and behaviors of urban AI/AN girls through data gathered from the 2002 National Survey of Family Growth (Rutman, Taualii, Ned, & Tetrick, 2012), findings revealed that approximately 33% of urban AI/AN girls were 15 years or younger at first intercourse compared with 14% of girls overall (Finer & Philbin, 2013). Of those urban AI/AN youth engaging in sex, 38% report having unprotected sex at first intercourse and 61% reported not using any method of contraception at the time they were interviewed (Rutman et al., 2012). By comparison, for the national sample, the median age at first intercourse never fell below 17 years and only 15% of those who were 17 to 18 years of age at first intercourse reported having unprotected sex (Finer & Philbin). These disparities highlight the need for teen pregnancy prevention interventions to address the specific needs of urban AI/AN youth. Although we were unable to find similar data to compare with AI/AN youth living on reservations, the findings of Kaufman et al. (2007) suggest that Northern Plains AI/AN youth also face intense pressures to engage in sex at young ages. Given the scarcity of studies examining the sexual behavior of reservation AI/AN youth, there is a need for building a better understanding of the contextual factors underlying sexual decision-making for both urban and reservation AI/AN youth.

## EXISTING TEEN PREGNANCY PREVENTION PROGRAMS

It is widely recognized that culturally grounded prevention programs enhance program effectiveness (Brave Heart, Elkins, Tafoya, Bird, & Salvador, 2012; de Ravello, Tulloch, & Taylor, 2012; Kirby, 2002), and a number of efforts have been made across the country to build on the assets and strengths of AI/AN culture to improve the reproductive health of these youth (e.g., Native STAND, Sacred Beginnings, Circle of Life, Project Red Talon). While strides have been made in recent years to understand the contemporary sexual and reproductive health expectations, pressures, and norms that influence today's AI/AN youth and how these might be shaped by lived cultural experiences (e.g., Rink et al., 2012; Rushing & Stephens, 2012; Rutman et al., 2012), there is still a need for teen pregnancy prevention programs to not only examine context but also to be responsive to the specific needs and priorities of the communities and participants these programs work with (Harris & Allgood, 2009).

At the national level, evidence-based teen pregnancy prevention programs often lump AI/AN populations with other minority populations, suggesting that these programs may have similar effects on participants, regardless of race and/or ethnicity. One of the few examples of a culturally tailored program that has provided evidence of effectiveness for AI/AN youth is Native STAND, which was originally adapted from Students Together Against Negative Decisions (STAND), a peer education program developed for youth in rural Georgia (National Coalition of STD Directors, 2012). This comprehensive, skills-based program has been modified from its original version to better address the needs of AI/AN youth. The curriculum identifies and incorporates cultural strengths and traditional teachings coupled with the recognition that AI/AN youth face many of the same challenges as mainstream youth (National Coalition of STD Directors). Pre and post survey data from Native STAND indicates that student participants showed increases in sexual health communication with peers, knowledge of reproductive health and STI/HIV prevention, intention to use condoms to avoid pregnancy and STIs, and condom self-efficacy (de Ravello, Rushing, Doshi, Smith, & Tulloch, 2011). These findings indicate the necessity of prevention programs to be responsive to community and cultural needs while simultaneously focusing on sexual behavior, including contraceptive use.

Another evidence-based curriculum, the Discovery Dating curriculum, emerged as a result of the needs expressed by women of the Oneida Indians of Wisconsin reservation and is based on AI/AN teachings, traditions, and cultural norms as the foundation of healthy relationship development and sexual abstinence. Discovery Dating is a five-year teen pregnancy prevention program that was implemented with eighth-grade students attending a rural tribal school. Study findings indicate fewer pregnancies and higher rates of condom use among program participants compared to the control group; further highlighting the potential impact of culturally based approaches to support sexual health and reduce teen pregnancies (Hagen, Skenandore, Scow, Schanen, & Clary, 2012).

The *Live It!* program, another example of a culturally tailored, teen pregnancy prevention and sex education program, has two versions of the curriculum, one for AI/AN youth ages 12–18 and one for parents, guardians, and other adults who work with AI/AN youth. The program was developed by the Division of Indian Work in Minneapolis, MN with the intention that the program could be adapted by tribes. According to The National Campaign to Prevent Teen and Unplanned Pregnancy (2009), the program has been implemented with more than 700 youth and 90 adults in more than 60 states. Pre/post test results suggest the program has increased knowledge among program participants about puberty and healthy relationships.

## LIMITATIONS OF EXISTING PROGRAMS

As seen in these examples, a number of teen pregnancy programs have achieved measurable successes. However, various problems with disseminating such “model practices” within AI/AN communities have also been identified. In their response to the evidence-based program mandate, Walker and Bigelow (2011) proposed that such programs and practices are not always adaptable to AI/AN communities, monitoring and evaluation methods are often unfamiliar to AI/AN tribes, and the implementation of such programs is often seen as a threat to the loss of traditional practices and infringement on tribal sovereignty. To avoid these potential pitfalls, the authors suggest the importance of working with AI/AN communities to ensure that practices and programs are based on local community context and culture (Walker & Bigelow).

Given this need for teen pregnancy prevention programs to be responsive to the specific cultural needs and contexts of AI/AN communities, it is important that researchers and practitioners understand the needs and context of the communities and youth with whom they work. In our review of the literature, the types of pregnancy prevention programs and suggestions for cultural tailoring that are of interest to urban and reservation-based AI/AN youth were rarely specified with a couple of exceptions. One of these exceptions was a study conducted by Garwick, Rhodes, Peterson-Hickey, and Hellerstedt (2008), which describes the various teen pregnancy prevention recommendations of 148 AI/AN youth living in the Minneapolis/St. Paul metropolitan area through the use of 20 focus groups with girls and boys aged 13–18 years. Content analyses of the focus group data revealed the following themes: “show the consequences of adolescent pregnancy; enhance and develop more pregnancy prevention programs for AI/AN youth in schools and community-based organizations; improve access to contraceptives; discuss teen pregnancy with AI/AN youth; and use key messages and media to reach Native youth” (Garwick et al., p. 81).

Another exception was a study by Rushing and Stephens (2012) using data collected from the Native Youth Media Survey, a comprehensive literature review, and community-based participatory research (CBPR) activities with Northwest tribes. Their study outlined the following considerations for inclusion in designing culturally appropriate, technology-based sexual health interventions with AI/AN youth in the Pacific Northwest: gender-specific information and age-appropriate content, abstinence, holistic approaches, cultural materials, asset and skill-building, confidential dialogue with trusted adults, interactive features, and evaluation strategies. The recommendations outlined in these studies emphasize the need for culturally competent and relevant teen pregnancy prevention programs for AI/AN youth.

## PRESENT STUDY

Given that previous research indicate that Northern Plains AI/AN youth face intense pressures to engage in sex at young ages, as illustrated by Kaufman et al. (2007), coupled with the scarcity of studies examining the sexual behavior of this particular population, there is a need for building a better understanding of the contextual factors underlying sexual decision making of Northern Plains AI/AN youth. In addition, to our knowledge, there are no evidence-based teen pregnancy prevention programs specific to Northern Plains AI/AN youth, further highlighting the need for the development of a teen pregnancy prevention program specific to the needs of this particular population. As such, Northern Plains American Indian (AI) youth were selected as the focus of the present study. (For the remainder of this article, the AI acronym is used in place of AI/AN given that none of the participants in this study identified as being a member of any of the Alaska Native tribes.)

The purpose of the present study is to provide community-driven suggestions for the design and implementation of a teen pregnancy prevention program for urban and reservation Northern Plains AI youth. Determining urban and reservation site differences in program recommendations was also a primary focus of this study. Understanding the variations in the needs and desires of these populations is beneficial in determining how to best tailor the program to fit the specific needs of these populations. In addition, particular attention was paid to understanding the gender differences in program recommendations to provide insight into the variations in the priorities of females versus those of males, which can be used to shed some light on how to better modify prevention efforts to address the specific needs of both genders.

## METHODS

### Community Engagement

Throughout the development of this project, we used a CBPR approach, which can be defined as a partnership approach to research which equitably involves community members and researchers in all stages of the research process by means of contributing expertise and shared decision-making and responsibilities, including the selection of appropriate research design and methodology (Israel et al., 2008; Sahota, 2010; Thomas, Rosa, Forcehimes, & Donovan, 2011). Throughout the development of this study, the following CBPR principles outlined by Israel et al. (2008) were adhered to: the recognition of the communities involved as a unit of identity; identifying and building upon the strengths and resources within communities; facilitating a collaborative, equitable partnership in all phases of the research process; working toward the mutual benefit of all partners; disseminating results to all partners and involving them in the dissemination process; and development of a long-term commitment to sustainability. This approach was selected as the appropriate method for involving community members given that this method has been cited as having higher participation rates and higher likelihood of producing lasting change, especially when working with AI communities (Fisher & Ball, 2003; Noe et al., 2007; Stubben, 2001).

As discussed by Sahota (2009, 2010) and Fisher and Ball (2003), the CBPR approach prioritizes the needs of community members and actively involves community members in the on-going regulation of the research process. In alliance with these principles, community advisory boards consisting of AI individuals of various ages, genders, and backgrounds were consulted throughout the research process, including the development of data collection instruments, establishing appropriate incentives, and identifying target populations. Because tribal anonymity is central to the community partner agreement, the specific tribal communities involved are not identified in this article. Prior to conducting this study, all study procedures were approved by the institutional review boards of Sanford Research, the affiliate university, and by the local tribe through tribal resolution for the reservation site.

### Design

The data collection and analysis methods reflect a qualitatively driven mixed methods design. Qualitative research methods were chosen for this particular study to capture participants’ unique points of view and experiences in their own words (Patton, 2002). The use of qualitative methodology is especially important when working with AI participants, as cultural context is most easily revealed and understood through open-ended, qualitative research methodologies (Denzin & Lincoln, 2000; Israel et al., 2005; Mohatt et al., 2008). In addition, quantitative methods were used to augment the qualitative findings to provide an understanding of the significant variations in program recommendations between participants based on their residence in an urban versus reservation setting and self-identified gender (Greene, 2007). Through this methodological thoroughness, analyses focus on both describing emergent themes and outlining key differences.

### Data Collection

#### Community Partners and Participants

The specific community partners involved in this study were selected as a result of on-going, long-term relationships and the collective prioritization and shared commitment to reducing teen pregnancy. The two communities that participated in this study included one reservation site and one urban site situated in the Northern Plains. Youth parents and nonparents were targeted for recruitment given their direct experience in relation to the sexual risk behaviors and pressures faced by youth in these communities and the application of results in the development of a teen pregnancy prevention program specific to the needs and context of this specific population. Elders were also selected for participation in this study because traditionally elders are accorded a place of significant importance, honor, and respect among Northern Plains AI tribes (Kehoe, 1982). Lastly, personnel at health care service facilities and schools with a high representation of AI patients and students, respectively, within both communities were recruited for participation in this study due to their familiarity with this population.

Data were collected by means of 24 focus groups (12 per site) with Northern Plains AI youth (both parents and nonparents) and elders and 20 semi-structured interviews (10 per site) with health care providers and high school personnel. A total of 185 community members participated in this study; however, two female parents from the reservation site who participated in the focus group did not complete the demographics survey. Of these participants, 90 were recruited from the reservation site and 95 were recruited from the urban site (see Table 1). Local community research associates recruited participants by means of flyers, community contacts, and word of mouth. Eligibility criteria for research participants in the focus groups included self-identification as AI and age. Specifically, youth were between the ages of 16 and 24 years, and elders were ages 50 years or older. For the interviews, key informants were identified through key community contacts and were not required to identify as AI, but were required to work directly with AI patients and students. Although age was not included in the eligibility criteria for participation in the key informant interviews, interviewees tended to be middle-aged (over the age of 40).

**Table 1:** Demographic Characteristics of Study Sample (*N* = 183)

		Age^c^	Gender^d^		Race^e^		
Method	*n*	mean (range)	Female	Male	AI/AN (alone)	AI/AN (multiple)	White (alone)
**Focus Groups (24 total)**^a^	**163**	**33.5 (15–79)**	**91 (55.8)**	**71 (43.6)**	**135 (82.8)**	**24 (14.8)**	**2 (1.2)**
Youth (Nonparents)	48	18.7 (15–24)	26 (54.2)	22 (45.8)	36 (75.0)	9 (18.8)	2 (4.2)
Youth (Parents)^b^	57	21.8 (17–25)	34 (59.6)	22 (38.6)	44 (77.2)	13 (22.8)	—
Elders	58	58.5 (44–79)	31 (53.4)	27 (46.6)	55 (94.8)	2 (3.4)	—
**Interviews (20 total)**^a^	**20**	**48.4 (28–69)**	**19 (95.0)**	**1 (5.0)**	**9 (45.0)**	—	**10 (50.0)**
School Personnel	10	47.5 (28–69)	9 (90.0)	1 (10.0)	5 (50.0)	—	4 (40.0)
Health Care Providers	10	49.3 (31–59)	10 (100.0)	—	4 (40.0)	—	6 (60.0)

^a^The focus group and interview data in bold represent the cumulative data for these two methods of data collection.^b^Two female parents did not complete the survey.^c^Three participants from the elder focus group did not report their age.^d^One participant from the parent focus group identified as both male and female and gender is represented as missing.^e^One participant from the youth focus group did not report their race. One participant from the elder focus group identified as Hispanic only. One of the school personnel interviewed did not report their race.

Data collection began during fall 2012 and continued through the winter and spring 2013. The focus groups and interviews were conducted in private rooms at local community health service buildings or libraries at each site. Focus groups included 5–11 participants per group, and groups were stratified by age, gender, and parental status. Written consent or assent was obtained from all participants, and parental consent was obtained for participants younger than 18 years of age. Participants were offered a $40 gift card to a retail store for their participation in the interview or focus group. The interviews and focus groups were tape-recorded and transcribed verbatim.

### Analytic Procedures

#### Qualitative Methods

Focus group and interview questions focused on soliciting recommendations for getting youth interested in participating in a teen pregnancy prevention program, program content, and where and when to host the program. In addition, interviewees were asked what the community's role is, if any, in preventing teen pregnancy. Responses to these questions contributed to detailed recommendations on how to best tailor a teen pregnancy prevention program to fit the needs of urban and reservation-based Northern Plains AI youth.

Focus group and interview transcripts were stored and analyzed using the Qualitative Solutions and Research International NVivo 10 software program (NVivo, 2013). As recommended by Fonteyn, Vettese, Lancaster, and Bauer-Wu (2008), NVivo was also used to create an electronic database of the transcripts that had been coded using the consensus codebook, as well as to update and revise the evolving codebook. Data were analyzed using inductive content analysis in which themes were uncovered through reading all transcripts, making notes on initial impressions, and letting the codes emerge directly from the text (Hsieh & Shannon, 2005; Ryan & Bernard, 2000). Recurring team meetings were held for in-depth discussion of the various topics that emerged and the negotiation of how to best translate these topics into definable and measurable themes, or codes, as well as the coding structure as it evolved throughout the analysis of transcripts.

The development of the codebook was guided by recommendations from MacQueen, McLellan, Kay, and Milstein (1998), in which codes were operationalized through coding definitions and coding decision rules in an iterative process involving multiple coding manual revisions. This process involved the systematic review of codes by two independent coders to determine the utility of the codes and consistency in their application. Specific sections of unique text were coded by the two coders independently, and inconsistencies were reviewed by the coders and the principal investigator. Disagreements among coders with respect to the interpretation and application of codes and/or coder errors were identified and discussed until a consensus was reached on how to best define codes and set parameters for coding text. In such cases, the codebook was clarified as needed. All previously coded text was then reviewed and, if necessary, recoded to maintain consistency with revised definitions and/or application parameters. This process ultimately resulted in 42 coding categories, which is not unusual given the amount of data gathered by means of the 20 interviews and 24 focus groups.

#### Quantitative Methods

After the themes were determined through the extensive qualitative analysis described above, frequencies were calculated for each theme and subsequent subthemes using SPSS version 20. Chi-square analyses were used to determine whether there were significant differences between the expected frequencies and the observed frequencies in the distribution of subthemes (e.g., categories of recommendations for program content and activities) between urban and reservation sites and females and males. The number of categories selected for this test was restricted to assure that less than 20% of the minimum expected cell frequency was less than 5. The traditional Bonferroni method of post-hoc analysis was also run to test for which specific differences contributed to the overall difference observed in the chi-square tests. This particular method of post-hoc analysis was selected given its ability to make multiple comparisons while simultaneously correcting for the significances of each comparison. Both chi-square and post-hoc results were considered significant when *p* <.05.

### Reliability and Validity

Another important piece of the qualitative data analysis was the calculation of inter-rater reliability. Two coders separately coded a random selection of 300 lines from the transcripts using the final draft of the codebook as recommended by Lombard, Snyder-Duch, and Bracken (2010). The kappa value was considered “substantial” (0.61 to 0.80) using the benchmarks set by Landis and Koch (1977).

Additional validity and reliability of the qualitative data were established through various methods. For example, ontological appropriateness and contingent validity were strengthened through the use of a diverse range of participant perspectives to describe the reality of teen pregnancy among AIs (Healy & Perry, 2000). Descriptive validity was strengthened through the use of verbatim responses and investigator triangulation, which was obtained by means of cross-checking coding schemes to ensure that the investigators agreed on the categorization of the data (Johnson, 1997; Maxwell, 1992). Interpretive validity, which refers to the accuracy in which the researchers portrayed the meaning attached to the data as perceived by the participants (Johnson; Maxwell), was also strengthened through the use of verbatim responses in that little was left up to interpretation outside of the creation of categories in which the verbatim responses were coded (Johnson; Maxwell). The results and discussion relate closely to the actual written responses of the participants and would, therefore, be deemed as having strong theoretical validity.

## RESULTS

Analysis of data resulted in six categories under program recommendations: target population, timing, location, program staff, incentives, and content/activities. The suggestions which fell under the first five categories of target population, timing, location, program staff, and incentives are consistent with those found in similar studies (e.g., Garwick et al., 2008; Rushing & Stephens, 2012) which stressed the importance of a school-based program for elementary and middle-school aged youth, facilitated by dedicated, respected, and trustworthy AI individuals from the community that youth could relate to. In addition, the most common suggestions for incentives to get youth interested in participating in the program included food and gift cards. The remaining results provide a more in-depth focus on recommendations for program content and activities, given its direct implications for the cultural tailoring of the curriculum.

### Content and Activities

When asked what participants would like to see included in a teen pregnancy prevention program, responses fell within 21 categories of suggested program content and activities (see Table 2 [Table T0002A] for a complete list of categories with *n* > 5 and example quotes). The top five categories (detailed further below) were testimonials/guest speakers, cultural education, hands-on activities, sex/reproductive health education, and teen pregnancy impact. A summary of recommendations for a strengths-based approach to curriculum design is also included even though this recommendation was less frequent than a number of other categories given that such suggestions were characteristic of differences in program recommendations by site.

**Table 2:** Suggestions for Program Content & Activities (*N* = 392 recommendations)

Response^a^	N (%^b^)	Example responses
Testimonials/Guest Speakers^c^	62 (15.8)	“Yeah, when I was little, whenever we used to have people come to assemblies and stuff, that would really catch my eye. ‘Cause like, for one thing, you’re getting the kids out of class, and you have all the kids’ attention.” – urban male youth (nonparent)
Cultural Education^d^	52 (13.3)	“I think getting back to our culture, cultural ways.” – reservation male elder
Hands-On Activities	43 (11.0)	“Just like a bunch of activities, like everybody said, different activities, games.” – urban male youth (parent)
Sex/Reproductive Health Education	41 (10.5)	“I think the schools, if they were more consistent in delivering and helping provide sexual health in an open, honest discussion, that there might be some changes.” – reservation health care provider
Teen Pregnancy Impact	33 (8.4)	“Educate them on what they could do if they didn't have a kid in high school compared to what they would do if they did have a kid, and how much difference it would make in their life.” – reservation male youth (nonparent)
One-On-One Setting	25 (6.4)	“It can start off one-on-one, and then maybe…” – reservation female youth (nonparent)
Contraception	22 (5.6)	“… like how to use contraceptives and teach them about contraceptives, because I didn't know about like Plan B until after I was pregnant. It would have been nice to know.” – reservation female youth (parent)
Group Setting	17 (4.3)	“I think these focus groups are good for young people.” – reservation female elder
Empowerment/ Self-Esteem	17 (4.3)	“You know, for the women, just show that, you know, they have respect for their body. You know, that it's not something that needs to be, you know, you don't have to do it, have sex, to be liked or loved or anything like that.” – reservation school personnel
Healthy Relationships	13 (3.3)	“How they can strengthen their relationship with the baby or maybe just start from there. You can have a baby and build a relationship with the father, the mother, or the grandparent, and start with that.” – urban female elder
Values/Morals	13 (3.3)	“I think our boys just need to learn to, well one, they need to learn to respect women.” – urban school personnel
Strengths-Based Approach	9 (2.3)	“Make it a positive outlook for them. Don't let them see their child as a regret, because that is the worst thing that can happen.” – reservation female youth (nonparent)
Youth Input	8 (2.0)	“Maybe ask them what they would like to see. Give them a survey, just like a piece of paper of what they would like to do at their age, high school, middle school, what they would like in a program. Ask them instead of just having this program all planned out, ‘This is what you’re going to do.’” – reservation female youth (parent)
*(Continued on next page)*

**Table NaN T0002A:** Suggestions for Program Content & Activities (*N* = 392 recommendations) *(Continued)*

Response^a^	N (%^b^)	Example responses
Humor	7 (1.8)	“In the future, you can't get pregnant without a permit.” – urban male youth (parent)
Miscellaneous	6 (1.5)	“Get the mental health…” – urban school personnel
Media	6 (1.5)	“Find some medium that they understand. Like media's a big thing that teaches them how, you know, it's okay to do it. I mean, if the media would just reverse themselves now, but that doesn't sell.” – reservation male elder

^a^Themes with less than five responses were not included in the table.^b^Percent of responses that fall into this category.^c^One individual indicated a desire not to include testimonials/guest speakers in the program.^d^Six individuals indicated a desire not to include cultural education in the program.

#### Testimonials/Guest Speakers

The most frequent program content and activities suggestion was to include testimonials and/or guest speakers (*n* = 61) primarily from current or former teen parents. A young, urban father who suggested that a teen parent come in as a guest speaker commented, “Tell them their own experiences, bad or good, or good or bad, you know. Especially like the bad experiences, I think that would have a lot of effect on the youth… .” Other suggestions for testimonials and guest speakers included storytelling. A reservation elder expressed his want for storytelling as follows:

I think one of the things that, I guess, I would like to see, or have seen, would be our older elders to share their stories with not only their grandchildren, but maybe within our tribal school system, because there's a lot of knowledge there. And these kids, if you share a story with them about your personal life, a lot of times they’ll open up to you. And I think our elders need to do that.

Overall, suggestions that fell under this category reflect a desire for the program to highlight real, lived experiences from individuals who can share the wisdom they have gained from experiencing life's struggles.

#### Cultural Education

The second most common recommendation was for program content to have a cultural focus (*n* = 47) through the incorporation of traditional, cultural teachings. It should be noted that six responses articulated hesitations about including cultural education in the program, all of which were from reservation study participants, either because some youth are not involved in traditional ceremonies, are not sure how cultural education relates to sexual decision-making, or because the school system may resist. However, these responses were coded under the overall cultural education category.

One urban youth (nonparent) summed up her desire to include culture in the following way:

I think, as far as the Native American part goes, I think if people were more educated about their culture and like traditions, and how things used to be, and what should be carried on today, maybe they would focus more on that. And like, and maybe that would become more time-consuming and like more of like a value too than, rather than choosing to be sexually active.

A similar sentiment was expressed by a reservation elder in her explanation for why including cultural education is important. She said, “Because within our ceremonies and our culture, you know, there was [*sic*] ways that we taught, you know, peoples [*sic*] who, our women and our men, what their roles and responsibilities were.”

Some of the specific ways offered in how to best incorporate culture were the inclusion of tribal language, history, cultural/ethnic pride, ceremonies, and attending powwows as well as the incorporation of traditional teachings, skills, and values with respect to how to be a healthy man or woman and develop healthy relationships, especially with family. The use of traditional cultural immersion camps or workshops was also suggested. For example, one reservation male elder recalled:

I've seen a couple of things this summer where different reservations have these kind of ceremonies for these girls that are reaching a certain age, going from puberty to the next step, and they kind of take them through a ceremony. I think that's pretty cool. I mean, I think something like that. You could use that to talk about all these kinds of things, like we were talking about tonight, to just help them through the rough spots, you know? Tell them what's important, how they should be, how the boys should treat them, and raise the bar a little bit for themselves.

Although the types of teachings, skills, and values were rarely discussed, the value of respect was mentioned a number of times, in particular, by teaching men to respect women. As one reservation school personnel stated, “… you just got to show that respect for the women. That's what our culture goes back to is that utmost respect for women, and don't treat them as objects.”

#### Hands-On Activities

The third most common suggestion was to include hands-on activities in the program (*n* = 43). Many of these recommendations reflected experiential and kinesthetic learning techniques, in which the process of creating meaning is derived from direct experience. Included in this was the idea that involvement in athletics often acts as a deterrent to engaging in sexual risk-taking behavior. As one male youth (nonparent) living on a reservation suggested, “The sports, it could show ‘em [*sic*] like how hard it would be to stay in sports with a kid.” Similarly, one urban school personnel discussed how keeping youth actively involved in various activities might be an effective risk-taking behavior prevention method. She said, “… I guess that's another thing, kind of, you know, with just that prevention piece, is keeping kids active so they’re not having nothing [*sic*] to do and searching for things and [*sic*] that they maybe shouldn't be involved in.” Another way that incorporating hands-on activities was mentioned was in terms of making the program more appealing and fun for youth. This sentiment was reflected in a comment by a reservation elder. He stated, “First of all, you have to bring it down to their level. Make it interesting. Make it fun.”

#### Sex/Reproductive Health Education

The fourth most frequently mentioned suggestion was to include sex/reproductive health education (*n* = 41). This recommendation often emphasized the need for providing youth with accurate, comprehensive information about sex and reproductive health so as to provide youth with the knowledge and the resources to make healthy, informed sexual decisions. An urban health care provider voiced this concern in the following way, “Then there's the blatant reality that kids need to know what their options are and certainly the health risks of STDs and everything. So I believe, totally, in being very open with kids… .” Respondents often indicated that conversations with youth about sex, regardless of whether these conversations are initiated by parents, teachers, or health care providers, should be open and honest. As one reservation health care provider stated, “I think having a conversation and listening to, and just being able to talk openly with the younger people about it. Just ask the questions, not being ashamed or afraid.”

#### Teen Pregnancy Impact

The fifth most recurrent suggestion for program content and activities was to highlight the potential impact of teen pregnancy on a person's life (*n* = 34). Similar to suggestions for having teen parents as guest speakers, recommendations that fell under this category often reflected the need for program content to include factual information which relate to lived experiences reflecting the potential hardships of teen parenting. As one urban male youth (nonparent) stated, “Yeah, honestly, that's the way I really think kids need to be taught these days. Get it in their face. Be straight up. Say it how it is. Tell ‘em [sic] how it is to struggle with a kid… .” In her suggestion to highlight the financial responsibility of raising a child, one female youth living on a reservation commented, “And I think, like, if teens knew the cost of raising a baby, they would be more careful or, I don't know, more interested in preventing themselves from having a baby.” In addition to including the cost of raising a child, other suggested impacts included: how a person's body changes throughout pregnancy, labor and delivery, potential difficulties in completing high school and pursuing postsecondary education, and the daily challenges of raising a child.

#### Strengths-Based Approach

Suggestions for a strengths-based approach to curriculum design were the 12th most recurrent theme. Consistent with the underpinnings of a strengths-based philosophy to pedagogy which draws upon the unique strengths, resources, and capacities of individuals and communities as potent sources of self-determined change (McCashen, 2005), such recommendations often highlighted the need to abandon deficits-based approaches in favor of one based on strengths and resilience. For example, a female elder living on the reservation explained, “… we get just so sick of hearing all these negative things. We lead in the uh, you know, worst statistics. Well, okay, we know that, but let's not, let's go at it in a different way.” Similarly, one reservation-based female youth (nonparent) stated, “Make it a positive outlook for them. Don't let them see their child as a regret, because that is the worst thing that can happen.” Other suggestions that fell under this category articulated the need to build upon the strengths and resources available to youth as a means of promoting healthy decision making.

### Differences by Location

To perform a more in-depth analysis of the data patterns, a mixed methods approach was used; namely, chi-square independent tests were run to test for significant differences between program recommendations and residence in an urban versus reservation area. A significant overall difference was found between program content and activities (top 12 subcategories) and area of residence (chi-square = 35.8; df = 11; *p* <.001). Specifically, post-hoc analyses comparing column proportions and adjusting the *p* values (Bonferroni method) revealed a significant difference in the frequency in which recommendations for including sex/reproductive health education were given by residency, with such recommendations occurring more often among the urban participants (27 vs. 14). A difference was also observed in the frequency in which recommendations for incorporating hands-on activities and utilizing a strengths-based approach were given by residency, with both suggestions being more frequent among reservation-based participants (hands-on activities = 34 vs. 9; strengths based approach = 8 vs. 1). There was no significant difference between the remaining program content categories by area of residence.

### Gender Differences

Chi-square independent tests were also run to test for significant differences between program recommendations and gender. Analyses revealed a significant overall difference between program content and activities (top 11 subcategories) and gender (chi-square = 35.7; df = 10; *p* <.001). Notably, Bonferroni post-hoc analyses revealed a significant difference in the frequency in which recommendations for including sex/reproductive health education, cultural education, empowerment/self-esteem, and having one-on-ones were given by gender, with a higher frequency for all among females (sex/reproductive health = 36 vs. 5; cultural education = 27 vs. 19; empowerment/self-esteem = 16 vs. 1; one-on-one setting = 23 vs. 2). A difference was also observed in the frequency in which recommendations for incorporating hands-on activities were given by gender, with a higher occurrence of these recommendations among males (23 vs. 20). Although this difference might appear minimal at first glance, it should be noted that, overall, there were 230 program content and activities suggestions from females versus 93 from males.

## DISCUSSION

Using input from focus groups with AI youth and elders as well as key informant interviews with health care providers and school personnel, the goal of the present study was to garner community-driven recommendations for the development of a teen pregnancy prevention program to fit the needs and context of urban and reservation-based Northern Plains AI youth. The program recommendations described above provide detailed community prescriptions for content and activities. In sum, these results contribute toward the call for community-driven program recommendations outlined in previous studies (e.g., Brave Heart et al., 2012; Harris & Allgood, 2009; Walker & Bigelow, 2011). These recommendations will be used to advise the development of a school-based program curriculum for use in the urban and reservation-based Northern Plains AI communities involved in this study.

Similar to the findings of Rushing and Stephens (2012) and Garwick et al. (2008), these program recommendations characterize the importance of culturally tailoring the program for these populations with a particular focus on providing AI youth with the wisdom gained from lived experiences through the use of traditional storytelling and the application of traditional values, teachings, and ceremonies through curriculum content and activities similar to those used in the Native STAND, Discovery Dating, and Live It! programs. Unlike previous research findings outlining various AI teen pregnancy prevention program recommendations, study participants suggested utilizing experiential learning techniques by means of hands-on activities, which have been found to be effective learning techniques when working with AI youth, in general (O’Connor, 2009; Ratsoy, 2011). Recommendations focusing on providing accurate, comprehensive sexual and reproductive health information were also similar to the findings of Garwick et al. (2008) and the curriculums used in the Native STAND and Live It! programs.

Chi-square and post-hoc analyses revealed differences in program recommendations between urban and reservation site participants in terms of program content and activities. The urban site participants recommended sex/reproductive health education more often than reservation site participants, whereas the reservation site participants more regularly recommended incorporating hands-on activities and utilizing a strengths-based approach. This finding could be reflective of variations in cultural influences in that urban site participants could potentially be more assimilated to the majority culture compared with reservation site participants, who may align more with traditional AI culture. This can be seen in the fact that the use of experiential learning techniques are more often associated with traditional AI learning styles (O’Connor, 2009; Ratsoy, 2011), and the utilization of strengths-based approaches has been highlighted by other researchers in relation to its relevance to AI youth (Brownlee, Rawana, MacArthur, & Probizanski, 2010; McMahon, Kenyon, & Carter, 2013; Stiffman et al., 2007).

By comparison, the preference for sex/reproductive health education among urban site participants is more in line with recommendations for effective teen pregnancy prevention for the overall population, which often suggests addressing appropriate sexual risk-taking behaviors and contraceptive use as effective sex education techniques (e.g., Kirby, 2002; Manlove et al., 2002). In other research, Marsiglia, Nieri, and Stiffman (2006) found that involvement in cultural activities, such as family and individual involvement in AI traditions and ceremonies, was low among their sample of urban AI youth. These findings are also consistent with those of Garwick et al. (2008) in which urban AI youth often expressed the need and desire for comprehensive sexual health education in schools which suggests the possibility that urban AI youth, in general, are often more assimilated to mainstream culture compared to reservation AI youth.

As previously mentioned, analyses also revealed differences in program recommendations between females and males in terms of program content and activities, in which females more often suggested the inclusion of sex/reproductive health educational content, cultural content, increasing feelings of empowerment and positive self-esteem, and one-on-one counseling. By contrast, males more frequently suggested the inclusion of hands-on activities such as basketball tournaments as a mechanism for goal setting. This finding is supported by other analyses of this data (Hanson, McMahon, Griese, & Kenyon, 2014), which reveal that females often felt pressured by family to have children at a young age and often felt obligated to engage in sexual intercourse “to please their boyfriend, even if they don't want to” (p. 812). Although contraception use is often seen as the female's responsibility among both males and females alike (Hanson et al., 2014), females often felt a lack of empowerment in both the refusal of sex and negotiation of condom use. Contributing to males’ desire to include hands-on activities is the fact that a number of these youth have role models who are athletes or relatives who excelled at sports (Hanson et al., 2014). In particular, basketball is often a popularized sport in AI cultures and is often featured in AI magazines and novels (e.g., Donahue, 1997; Hudetz, 2014).

In relation to the research methods used in this particular study, such a considerable sample size (*n* = 185) is unusual in qualitative research. With a sizable dataset such as this, analyses were able to quantify the qualitative data to make statistical comparisons. The use of a mixed methods approach has proven to be beneficial in describing the program recommendations of study participants while also determining the specific site and gender differences in these recommendations. The use of a mixed methods design revealed both in-depth, contextualized program recommendations and statistically significant comparisons between participants based on the outcomes of interest. Determining site differences in program recommendations was important for understanding the variations in the needs and wants of these populations. In addition, understanding the gender differences in program recommendations provides insight into the priorities of females versus those of males, which can be used to better modify prevention efforts to address the specific needs of both genders.

The current data is being used to inform the development of an evidence-based, culturally appropriate teen pregnancy prevention program for urban and reservation-based Northern Plains AI youth called *My Journey.* Specifically, the needs assessment data is being used to determine the appropriate ages to target for participation in the program and the potential locations within the communities for implementation. The program includes both male and female youth, and the incorporation of the needs of both genders was given due diligence. Given the recommendation to provide comprehensive sexual and reproductive health education, the curriculum covers the changes that occur throughout puberty, healthy relationships, reproductive health and STI/HIV prevention, contraceptive knowledge and behavior, including contraception access and use, and particular attention is paid to incorporating communication modeling, negotiation, and refusal skills.

The program is guided by positive youth development strategies, which are consistent with suggestions for the use of strengths-based approaches, and works toward promoting empowerment in sexual health decision making. Lessons also incorporate hands-on activities to engage students throughout as suggested. To address the cultural needs and wants of these populations, the curriculum includes traditional AI teachings (e.g., the four quadrants of the medicine wheel), storytelling, and the application of traditional Northern Plains AI values (e.g., respect, humility, compassion, bravery, generosity, wisdom) to healthy decision making and discussions of local coming-of-age ceremonies. In adherence with suggestions to include testimonials, guest speakers, and to highlight the impact of teen pregnancy, vignettes are included to incorporate the wisdom of tribal elders and a former AI teen parent is an invited guest speaker to reflect on their experiences and struggles as a teen parent. Given the differences in the recommendations between urban and reservation community members, there are variations by site in determining which lessons are essential to program delivery and dosage, with those lessons relating to sex/reproductive health content being more of a focus at the urban site, while those lessons utilizing experiential learning techniques being more critical at the reservation site.

Moreover, the findings highlight the importance of taking into account indigenous methodologies and ways of knowing. As illustrated by Cochran et al. (2008), *how* knowledge is acquired is as important, if not more important, than the knowledge gained, especially in efforts to eliminate or reduce health disparities. Therefore, community involvement and cultural consultation are utilized throughout program and curriculum development and implementation to assure that traditional learning styles are being implemented accurately and appropriately. Given that the development of this program is guided by community-driven recommendations, the project will continue to involve AI community members, and we anticipate that the *My Journey* curriculum will have a sustained impact on Northern Plains AI youth.

### Limitations

Participants in the focus groups and many of the key informant interviews were primarily Northern Plains AIs; therefore, the results cannot be generalized to all AIs. Furthermore, quantitative analyses could not be run to compare differences in program recommendations between youth parents and nonparents, elders, school personnel, and health care providers because of the low *n* of the various coding categories. Future research could address these differences between the perceived need and desired characteristics of a teen pregnancy prevention program based on age, parental status, and community role. In addition, this research could be extended to other tribes and AI populations to explore differences in sexual decision-making and recommendations for teen pregnancy prevention efforts as they relate to specific AI cultures, gender norms, and variations in cultural assimilation.

## CONCLUSION

Our research advances the literature by providing detailed recommendations for the development of a teen pregnancy prevention program specific to the wants and needs of urban and reservation Northern Plains AI youth. The findings outlined in this article provide an in-depth look at how teen pregnancy prevention efforts might tailor a program to fit the wants and needs of these populations, while also outlining potential reasons for observed differences in the recommendations offered between urban and reservation sites and between females and males. Given that little is known about how to best tailor a teen pregnancy program to fit the cultural needs of these populations, this contribution adds to the literature by detailing the various community-driven suggestions for how this might be done. It also highlights the desirability of a culturally grounded teen pregnancy prevention program among both urban and reservation-based Northern Plains AIs.

## ACKNOWLEDGMENTS

The authors thank Dr. Renee Seiving, Melissa Huff, Char Green, Jen Prasek, Dr. Paul Thompson, Ashley Miller, Noelani Villa, Donna Keeler, Reggan LaBore, Kathy White, Cassandra Crazy Thunder, Tonya Belile, Chad Birger, and Ashley Schwab for their contributions to this project.

## FUNDING

The authors disclosed receipt of the following financial support for the research, authorship, and or publication of this article: This work was supported by the National Institutes of Health, National Center on Minority Health and Health Disparities [P20MD001631-06]. The content is solely the responsibility of the authors and does not necessarily represent the official views of the National Center on Minority Health and Health Disparities or the National Institutes of Health (NIH).
